# Cholinesterases in Tripartite Neuromuscular Synapse

**DOI:** 10.3389/fnmol.2021.811220

**Published:** 2021-12-23

**Authors:** Konstantin A. Petrov, Svetlana E. Proskurina, Eric Krejci

**Affiliations:** ^1^Arbuzov Institute of Organic and Physical Chemistry, FRC Kazan Scientific Center of RAS, Kazan, Russia; ^2^CNRS, Université de Paris, ENS Paris Saclay, Centre Borelli UMR 9010, Paris, France

**Keywords:** acetylcholinesterase, butyrylcholinesterase, congenital myasthenic syndromes, neuromuscular junction, terminal Schwann cells

## Abstract

The neuromuscular junction (NMJ) is a tripartite synapse in which not only presynaptic and post-synaptic cells participate in synaptic transmission, but also terminal Schwann cells (TSC). Acetylcholine (ACh) is the neurotransmitter that mediates the signal between the motor neuron and the muscle but also between the motor neuron and TSC. ACh action is terminated by acetylcholinesterase (AChE), anchored by collagen Q (ColQ) in the basal lamina of NMJs. AChE is also anchored by a proline-rich membrane anchor (PRiMA) to the surface of the nerve terminal. Butyrylcholinesterase (BChE), a second cholinesterase, is abundant on TSC and anchored by PRiMA to its plasma membrane. Genetic studies in mice have revealed different regulations of synaptic transmission that depend on ACh spillover. One of the strongest is a depression of ACh release that depends on the activation of α7 nicotinic acetylcholine receptors (nAChR). Partial AChE deficiency has been described in many pathologies or during treatment with cholinesterase inhibitors. In addition to changing the activation of muscle nAChR, AChE deficiency results in an ACh spillover that changes TSC signaling. In this mini-review, we will first briefly outline the organization of the NMJ. This will be followed by a look at the role of TSC in synaptic transmission. Finally, we will review the pathological conditions where there is evidence of decreased AChE activity.

## Organization of the NMJ: Cholinesterases

The overall organization of the neuromuscular junction (NMJ) contains three partners (Slater, 2017; Jones et al., 2017; Alhindi et al., 2021): (1) a nerve terminal, i.e., axonal ends of a motor neuron that releases the neurotransmitter ACh; (2) endplate, i.e., a small domain of the muscle fiber opposite the nerve terminal that is morphologically and functionally specialized for the generation of the endplate potential (EPP) and triggering of action potentials (AP); (3) terminal Schwann cells (TSCs).

Synaptic transmission results in several sequential steps: (1) depolarization of the motor neuron membrane triggers the simultaneous release of dozens of vesicles filled with acetylcholine (Ach) and ATP; (2) synchronous activation of nicotinic acetylcholine receptors (nAChRs) clustered on the crest of the post-synaptic membrane that depolarizes the membrane and triggers an action potential; (3) termination of the action of ACh by hydrolysis with acetylcholinesterase (AChE) localized in the synaptic cleft and anchored in basal lamina *via* a collagen-like tail [collagen Q (ColQ)]. It should be noted that AChE anchored by ColQ is an enzyme that limits not only the lifetime of ACh in the synaptic cleft but also the spillover of ACh outside of the synaptic cleft.

**FIGURE 1:**
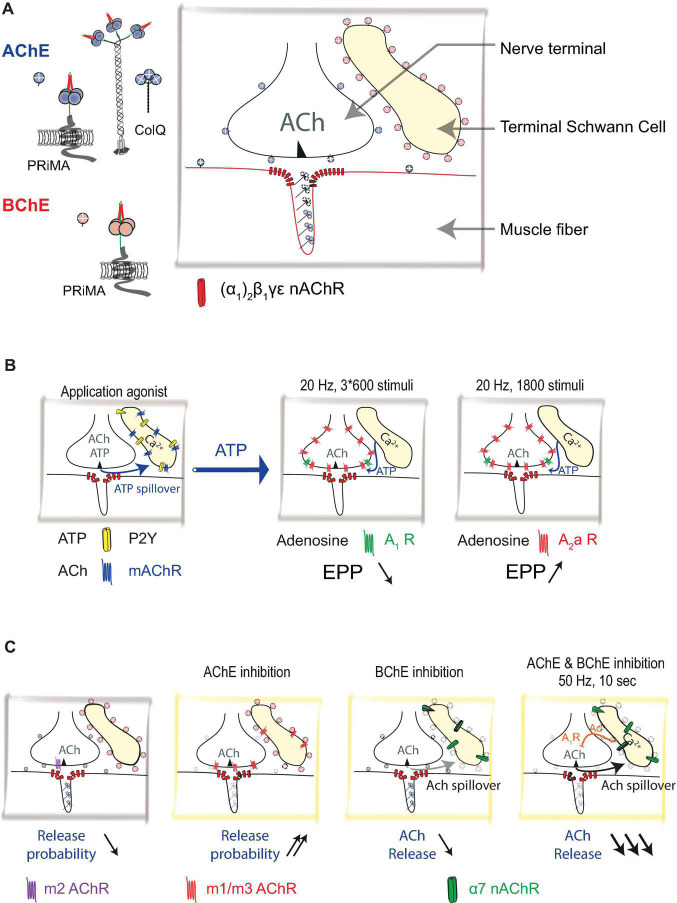
Distribution of acetylcholinesterase (AChE) and Butyrylcholinesterase (BChE) at mouse neuromuscular junction (NMJ): **(A)** Schematic representation of nerve terminal, muscle fiber, and terminal Schwann cell (TSC). Acetylcholine (ACh) is released from the nerve terminal. AChE is organized in complexes (collagen-tailed forms A12) see Figure 2. AChE/collagen Q (ColQ) are clustered in the basal lamina. AChE is also tethered by proline-rich membrane anchor (PRiMA) on the membrane of the nerve terminal. ColQ/AChE controls the activation of muscle-type nicotinic acetylcholine receptors [nAChR; (α1)2β1γε]. BChE is anchored at the TSC by PRiMA. **(B)** Calcium waves are triggered in TSC by muscarinic agonist or ATP. TSCs regulate ACh release by using ATP as a gliotransmitter. ATP released by the nerve terminal triggers calcium waves in TSCs *via* activations of P2Y receptors. These calcium waves cause the release of ATP from TSCs. ATP, degraded to adenosine, activates A1 and A2a receptors. If the A1 receptor response dominates (3 × 600 stimuli protocol), it results in a depression of ACh release, if the A2a response dominates (1,800 stimuli protocol) it results in the facilitation of ACh release. **(C)** ACh controls its own release by different mechanisms that are revealed using cholinergic agonists and cholinesterase inhibitors. The addition of muscarine (muscarinic AChRs agonist) results in a decrease in the probability of release by activation of m2 muscarinic acetylcholine receptors (mAChR). On the contrary, the inhibition of AChE results in an increase in the probability of ACh release by activation of m1/m3 receptors. Inhibition of BChE results in a very limited decrease of the ACh release *via* activation of α7 nAChRs. When AChE and BChE are inhibited, in 50 Hz 10 s protocol, ACh activates α7 nAChRs, triggers calcium wave in TSCs that release adenosine (Ad) as a gliotransmitter. ACh release is greatly diminished.

**FIGURE 2:**
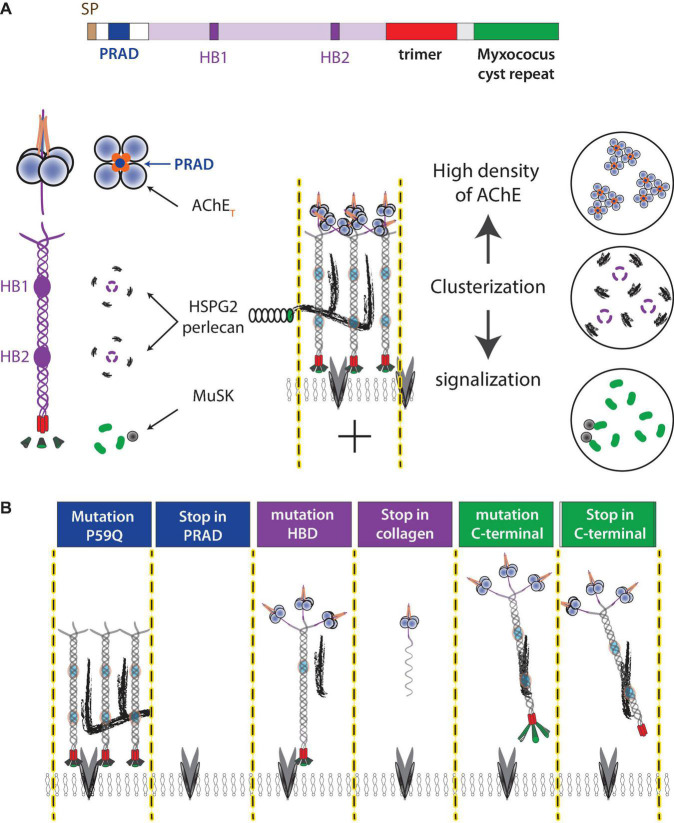
ColQ functions at NMJ. **(A)** ColQ is required to cluster AChE in the basal lamina and interacts with multiple partners. ColQ is a multidomain protein. A signal peptide translocates the single-stranded collagen subunit into the endoplasmic reticulum where the collagen trimer is assembled from the C-terminal domain. AChE tetramers are organized by a proline-rich sequence (PRAD) of each of the ColQ subunits. The mature complex thus assembles 12 catalytic AChE subunits. Four heparan sulfate chains [perlecan (HSPG2) and other proteoglycans] interact with two clusters of basic residues (heparin-binding site HB1 and HB2) in the trimer of collagen. The C terminal of ColQ interacts with MuSK and thus participates in NMJ signaling. **(B)** Mutations in the ColQ gene in humans are responsible for a congenital myasthenic syndrome with AChE deficiency. The mutations in the domains lead to different properties, which are illustrated in the diagram. For example, a point mutation (176C-A) changes P59 to Q (Ohno et al., 2000), kept the full-length ColQ intact but ColQ cannot interact with AChE, whereas 107del215 create a deletion of exon 2 encoding PRAD and the absence of ColQ (Ohno et al., 1998). Similar mechanisms occur in the heparin-binding domain where the mutation G237D (Mihaylova et al., 2008) should affect the interaction with heparan sulfate but keep intact ColQ, whereas R236X produces a shorter single-stranded collagen. Similarly, ColQ with mutations in the C-terminal domain organizes AChE into tetramer but AChE is not clustered in the basal lamina.

In mammals, AChE and butyrylcholinesterase (BChE) are enzymes that hydrolyze ACh and have similar molecular forms (Massoulié, 2002), both containing a catalytic domain and a similar C-terminal tetramerization domain that requires a proline-rich sequence to organize the tetramer (Figure 1A). Several peptides containing polyproline sequences were found in AChE and butyrylcholinesterase (BChE) tetramers (Li et al., 2008; Biberoglu et al., 2012, 2013; Schopfer et al., 2017), confirmed in the three-dimensional (3D) structure of BChE tetramer purified from the serum (Leung et al., 2018; Boyko et al., 2019); and two proteins ColQ and proline-rich membrane anchor (PRiMA) contain a proline-rich sequence that organizes AChE and/or BChE into tetramers. PRiMA is a small transmembrane protein that anchors AChE tetramer on the plasma membrane (Perrier et al., 2002; Dobbertin et al., 2009) and ColQ is specific collagen (Figures 1A, 2) that anchors AChE tetramers in the basal lamina (Feng et al., 1999). AChE is a hallmark of the mammalian NMJ, localized mainly in the basal lamina of the primary cleft between the nerve and the muscle fiber, and in the folds that penetrate the post-synaptic domain of the NMJ (Davis and Koelle, 1967; Salpeter, 1969; Anglister et al., 1994; Blotnick-Rubin and Anglister, 2018). Here, AChE is clustered by ColQ, and in fact, in the absence of ColQ there is a severe AChE deficit at the NMJ (see later).

The PRiMA/AChE that is presented at the NMJ can be produced by the motor neuron because in ColQ KO mice, which have only PRiMA/AChE, very fine staining of AChE was detected at the plasma membranes of the motor neuron but not at the surface of the muscle (Bernard et al., 2011). However, the PRiMA/AChE complex was extracted from muscle domains devoid of NMJ (Bernard et al., 2011) supporting that PRiMA/AChE is produced by the muscle, localized at the surface of the muscle, as previously proposed (Younkin et al., 1982), but the density is too low to be visualized with antibody or activity staining. It was proposed that membrane-anchored PRiMA/AChE can participate in ACh hydrolysis in the perisynaptic area (Dunant and Gisiger, 2017).

Butyrylcholinesterase (BChE) is also detectable at the NMJ (Davis and Koelle, 1967), more specifically BChE is anchored by PRiMA at the surface of TSC (Petrov et al., 2014).

These enzymes (AChE and BChE), localized and clustered in different compartments of the NMJ, efficiently eliminate ACh and thus prevent ACh spillover and limit the action of ACh to a single shot to the post-synaptic receptors.

## The Role of TSC in Synaptic Transmission

The main role of TSC in synaptic transmission is traditionally associated with controlling the concentration of K^+^ ions. When an action potential is generated, the accumulation of K^+^ ions released by nerve and muscle cells into the extracellular environment can lead to the depolarization of the cell membrane and consequent inactivation of Nav1.4 sodium channels. Thus, the timely removal by TSC K^+^ ions prevents the development of muscle fatigue (Heredia et al., 2018).

In addition to controlling the concentration of extracellular K^+^ ions, the release of Ca^2+^ ions from intracellular stores is observed in TSCs (so-called calcium oscillations). The short-term exogenous application of neurotrophin-3 or BDNF in newborn animals leads to an increase in the level of intracellular Ca^2+^ ions in the TSCs, which correlates with an increase in the release of ACh through the activation of presynaptic receptors of the tropomyosin receptor kinase (Trk) (Lohof et al., 1993). The concentration of Ca^2+^ in the TSCs can also change *via* G-protein associated receptors: the microinjection of GTPγS (a non-hydrolyzable analog of guanosine triphosphate) into TSCs reduces the induced release of the ACh, while the microinjection of GDPβS (a non-hydrolyzed analog of guanosine diphosphate) reduces the synaptic depression caused by high-frequency stimulation (Robitaille, 1998).

It has been shown that TSCs can influence ACh release from the motor nerve (Ko and Robitaille, 2015). Since a change in the amount of ACh secreted into the synaptic cleft primarily affects the amplitude of EPP, TSCs can change the safety factor of neuromuscular synaptic transmission.

It has been shown that an increase in intracellular Ca^2+^ in TSC (Figure 1B) gives rise to the secretion of ATP from the TSC (Robitaille, 1995), which, degrading to adenosine, activates the A1 and A2 subtypes of adenosine receptors (Todd et al., 2010). It is important to note that the TSC calcium response depends on the pattern of motor nerve stimulation. Long-term continuous stimulation (20 Hz, 1,800 stimuli) causes a relatively short-term increase in the level of Ca^2+^ in TSCs, which correlates with an increase in the EPP amplitude (Figure 1B); this effect is mediated by the activation of the A2a subtype of adenosine receptors. Stimulation of the motor nerve with three series of impulses at a frequency of 20 Hz for 60 s, having two intervals of 30 s between the series, correlates with a decrease in the EPP amplitude (Figure 1B). This effect is mediated by the activation of the A1 adenosine receptor subtype (Todd et al., 2010). Since neither post-tetanic potentiation nor post-tetanic depression is observed following injection of a Ca^2+^ chelator into TSCs, the role played by TSCs in modulating neuromuscular synaptic transmission through the balance between the activation of A1 and A2 subtypes of adenosine receptors can be seen as crucial. A similar mechanism has been described for newborn animals, in which there is a transition from polyneuronal to mononeuronal innervation of muscle fibers. It is important to note that less active motor nerve endings undergo elimination and that TSCs participate in the process of dividing competing synaptic contacts according to their level of activity. Thus, in detecting the level of synaptic activity through the P2Y1 subtype of ATP receptors, TSCs react with an increase in the level of intracellular Ca^2+^ to secrete additional ATP, which then comprises a source of adenosine. Activation of presynaptic A2a adenosine receptors leads to synaptic potentiation of the active motor nerve (Darabid et al., 2013, 2014, 2018).

It has been shown that inhibition of BChE, located on the surface of the TSCs, leads to a decrease in the amount of ACh released in response to the stimulation of the motor nerve (Petrov et al., 2014). This decrease is mediated by the activation of the α7 subtype of acetylcholine receptors (AChRs) (Figure 1C). It also has been shown that α7 AChRs controls tetanus-induced ACh spillover from the neuromuscular synapse by promoting adenosine outflow from TSCs *via* ENT1 transporters and retrograde activation of presynaptic A1 inhibitory receptors (Noronha-Matos et al., 2020). However, it is important to note that this pathway was activated by endogenous ACh only when AChE and BChE were inhibited by neostigmine (Figure 1C).

In response to the release of ACh, the release of Ca^2+^ from intracellular stores in TSCs triggered by the activation of muscarinic acetylcholine receptors (mAChRs) has been described (Robitaille et al., 1997; Rochon et al., 2001; Arbour et al., 2017). However, the effect of calcium oscillations in TSCs caused by the application of muscarine on ACh release remains an open question.

It is important to note that in general, the effect of the activation of mAChRs on ACh release in mammalian NMJs has been studied mainly in the presence of exogenous agonists (Abbs and Joseph, 1981; Wessler et al., 1987; Arenson, 1991; Robitaille et al., 1997; Santafé et al., 2003, 2004, 2006, 2007; Oliveira et al., 2002, 2009; Dudel, 2007; Kovyazina et al., 2010, 2015) or in conditions of AChE inhibition (Figure 1C). Thus, the physiological role of these pathways of autoregulation of ACh release is probably most pronounced under conditions of reduced AChE activity (Minic et al., 2002).

These mechanisms, demonstrated experimentally in the context of mature NMJ, are observable only after the inhibition of AChE and BChE. The depression of ACh release triggered by ACh spillover could be activated in physiological contexts where AChE density is significantly decreased. For example, during motor unit remodeling, some neuronal terminals are no longer connected to the muscle fiber that accumulates AChE and thus ACh spillover can be detected by TSC and thereby limit ACh loss. These mechanisms are useful during tissue remodeling and may accentuate pathological alterations in NMJ when the AChE level is reduced.

## Pathological Conditions With AChE Deficiency

To date, over 60 mutations have been identified in the human ColQ gene, all of which lead to a congenital myasthenic syndrome with endplate AChE deficiency (Ohno et al., 2000; Mihaylova et al., 2008; Wargon et al., 2012; Nakata et al., 2013). ColQ is a multifunctional protein encoded by a single gene. ColQ contains a short proline-rich domain (PRAD) that organizes the AChE tetramer. ColQ contains a collagen domain, folded in trimer with the help of a specific C-terminal domain that assists the folding of this complex oligomer (Bon et al., 2003). This 50 nm long collagen contains two different heparin-binding sites with different properties (Deprez et al., 2003). These sites contribute to the interaction with heparan sulfate proteoglycan as perlecan (Peng et al., 1999; Arikawa-Hirasawa et al., 2002). Interaction with heparan is necessary but not sufficient to cluster AChE. ColQ contains a Myxococcus cysteine repeat that interacts with muscle-specific kinase (MuSK), a member of a post-synaptic signaling pathway, which is responsible for AChR clustering (Figure 2; Cartaud et al., 2004; Sigoillot et al., 2010; Nakata et al., 2013). Despite the consistent severe AChE deficit at the NMJ, the severity of the disease is not correlated with the mutations, suggesting that other properties of ColQ may be involved. Two structural functions of ColQ may have significant effects on NMJ organization. The trimer of ColQ interacts with heparin and potentially with different heparan sulfate proteoglycans (HSPG) in the basal lamina, and the reorganization of the basal lamina in the absences of ColQ may contribute to different changes (Figure 2).

The C-terminal domain of ColQ interacts with the receptor tyrosine kinase MuSK, master organizer of NMJs (Sigoillot et al., 2010; Nakata et al., 2013), and counteracts the action of the agrin/LRP4 complex (Otsuka et al., 2015). During embryonic development, nAChR clustering is stabilized by the activation of MuSK by the agrin/LRP4 complex (Burden et al., 2013) and destabilized by Ach (An et al., 2010). In adults, LRP4/Agrin/MuSK and electrical activity are required for the maintenance of nAChRs (Witzemann et al., 2013; Martinez-Pena et al., 2021). Increased signaling by ACh may decrease the stability of nAChR clusters, a critical factor in maintaining synaptic transmission.

In addition to these post-synaptic mechanisms, the probability of ACh release is changed in ColQ KO mice (Figure 1C; Minic et al., 2002). During the tetanic activity, it is, therefore, possible that in the absence of AChE, the spillover of ACh triggers the depression of ACh by activation of α7 nAChR localized at the surface of the TSC. Depression of ACh release may become a major process to explain the transient fatigability that appears rapidly during physical exercise.

Around 5–15% of patients with myasthenia gravis carry autoantibodies directed against MuSK (Otsuka et al., 2015). It was shown that MuSK antibodies interfere with the MuSK-ColQ binding site that might reduce the synaptic AChE density (Kawakami et al., 2011).

The endplate AChE deficiency has been described in the mouse model of Schwartz-Jampel syndrome. It results from mutations of the gene encoding perlecan (Stum et al., 2008).

It was shown that AChE deficiency contributes to NMJ dysfunction in a mouse model of type 1 diabetic neuropathy (Garcia et al., 2012). Diabetic peripheral neuropathy is one of the most common complications of diabetes mellitus (Zenker et al., 2013), and responses of Schwann cells to diabetes-induced hyperglycemia are central to the pathogenesis of diabetic neuropathy (Mizisin, 2014). Thus, TSC may contribute to endplate pathology and subsequent muscle weakness during diabetes.

## Conclusion

In this mini-review, we have analyzed the function of cholinesterases in tripartite NMJ. In this light, AChE deficiency or inhibition results in an ACh spillover that can be detected by the TSC. Therefore, a better understanding of the molecular and cellular mechanisms of these communications could lead to the discovery of new possibilities for the development of therapeutic methods. On the other hand, the further study of pathological conditions associated with AChE deficiency could help to better understand the contribution of TSC to neuromuscular synaptic transmission.

## Author Contributions

All authors contributed equally to the writing and editing of the manuscript.

## Conflict of Interest

The authors declare that the research was conducted in the absence of any commercial or financial relationships that could be construed as a potential conflict of interest.

## Publisher’s Note

All claims expressed in this article are solely those of the authors and do not necessarily represent those of their affiliated organizations, or those of the publisher, the editors and the reviewers. Any product that may be evaluated in this article, or claim that may be made by its manufacturer, is not guaranteed or endorsed by the publisher.
